# Pharmacokinetics of Orally Administered Oseltamivir in Healthy Obese and Nonobese Thai Subjects

**DOI:** 10.1128/AAC.01786-13

**Published:** 2014-03

**Authors:** Podjanee Jittamala, Sasithon Pukrittayakamee, Joel Tarning, Niklas Lindegardh, Warunee Hanpithakpong, Walter Robert John Taylor, Saranath Lawpoolsri, Prakaykaew Charunwattana, Salwaluk Panapipat, Nicholas J. White, Nicholas P. J. Day

**Affiliations:** a Mahidol-Oxford Tropical Medicine Research Unit, Faculty of Tropical Medicine, Mahidol University, Bangkok, Thailand; b Centre for Tropical Medicine, Nuffield Department of Clinical Medicine, University of Oxford, Oxford, United Kingdom; c Faculty of Tropical Medicine, Mahidol University, Bangkok, Thailand

## Abstract

Oseltamivir is the most widely used anti-influenza drug. In the 2009 H1N1 pandemic, in which the influenza viruses were oseltamivir sensitive, obesity was identified as a risk factor for severe disease and unfavorable outcomes. The aim of this study was to investigate the pharmacokinetic properties of oseltamivir and its active metabolite, oseltamivir carboxylate, in obese and nonobese healthy subjects. A single-dose, randomized, two-sequence crossover study was conducted in 12 obese and 12 nonobese healthy Thai volunteers. Each volunteer was given 75 mg and 150 mg oseltamivir orally with an intervening washout period of more than 3 days. The pharmacokinetic properties of oseltamivir and oseltamivir carboxylate were evaluated using a noncompartmental approach. The median (range) body mass indexes (BMIs) for obese subjects were 33.8 kg/m^2^ (30.8 to 43.2) and 22.2 (18.8 to 24.2) for nonobese subjects. The pharmacokinetic parameters of oseltamivir carboxylate, the active metabolite of oseltamivir, were not significantly different between obese and nonobese subjects for both 75-mg and 150-mg doses. Both doses were well tolerated. Despite the lower dose per kilogram body weight in obese subjects, there was no significant difference in the exposure of oseltamivir carboxylate between the obese and nonobese groups. Standard dosing is appropriate for obese subjects. (The study was registered at ClinicalTrials.gov under registration no. NCT 01049763.)

## INTRODUCTION

Oseltamivir is a neuraminidase inhibitor widely considered the drug of choice for both the treatment and prophylaxis of influenza (1–4). Oseltamivir phosphate is a prodrug that is readily absorbed from the gastrointestinal tract and is then rapidly metabolized by the hepatic carboxyl esterase enzyme to its active metabolite, oseltamivir carboxylate (5, 6). The current recommended oral dose for influenza treatment for adults and older children is 75 mg taken twice a day for 5 days, irrespective of body weight (1, 2, 7). However, during the outbreaks of highly pathogenic avian influenza A/H5N1 virus, and also in the 2009 A/H1N1 pandemic, higher doses and longer durations of oseltamivir treatment were widely prescribed to patients with severe disease (7, 8, 9–13). Furthermore, the WHO and the U.S. CDC have published treatment guidelines that recommend the use of longer treatment courses in patients who remain severely ill after receiving oseltamivir treatment for 5 days and for immunocompromised patients (2, 3).

Obesity was an important independent risk factor for severe influenza and unfavorable outcomes identified during the 2009 A/H1N1 pandemic (2, 9, 13–18). However, there are few data on the pharmacokinetic properties of oseltamivir in obese patients. Ariano and colleagues reported a clinical pharmacokinetic study in critically ill patients with high body mass index (BMI) and with normal renal function in which the pharmacokinetic parameters of oseltamivir carboxylate were similar to those in healthy obese patients. The study found no correlation between body weight and drug exposure, as well as the volume of distribution of oseltamivir carboxylate, in severe 2009 A/H1N1 influenza (10). These data are also consistent with the findings of Pai and Lodise and of Thorne-Humphrey et al. in healthy obese Caucasian volunteers published recently (19, 20).

There has been an alarming increase in the rates of obesity in developing countries, particularly in Asia, where influenza pandemics typically originate (21–26). The aim of this study was to compare the pharmacokinetic properties of oseltamivir and oseltamivir carboxylate in obese and nonobese Thai subjects.

## MATERIALS AND METHODS

### Study design.

This was an open-label, crossover, randomized pharmacokinetic study conducted in 12 obese and 12 nonobese healthy adult subjects at one study center in the Faculty of Tropical Medicine, Mahidol University, Bangkok, Thailand. The study was registered at ClinicalTrials.gov under registration no. NCT 01049763. The subjects received single doses of 75 mg and 150 mg of oseltamivir in a random sequence for 2 visits with an intervening washout period of more than 3 days. The medical history was documented, and a physical examination was performed by study physicians before the study started. A complete blood count and clinical chemistry, including liver function tests, blood urea nitrogen, creatinine, electrolytes, blood glucose, serum lipoprotein, and triglyceride measurements, were performed at screening and before and 24 h after each drug dose. An electrocardiogram was performed at screening, predose, and at 4 h after drug dosing. A serum pregnancy test was done on screening before each admission. The use of contraception was advised throughout the study period and for 4 weeks after the last dose of drug. Adverse events were captured and graded based on the Division of AIDS table for grading the severity of adult and pediatric adverse events, version 1.0, of December 2004 with clarification in August 2009 (27).

A single dose of orally administered oseltamivir (Tamiflu; Hoffman La Roche) was taken in the fasting state (8 h before and 2 h after drug administration). Drug administration was directly observed by study personnel. Fluids were restricted to less than 3 liters/day during the 24 h after drug dosing.

Blood (2 ml) was taken for the pharmacokinetic analysis in fluoride-oxalate tubes at −30 (predose), 30, 60, 90, 120, and 180 min and then at 4, 5, 6, 7, 8, 10, 12, and 24 h after drug dosing. The blood samples were centrifuged for 7 min at 2,000 × *g* at 4°C. After centrifugation, the plasma was stored at −70°C or lower until analyzed.

### Sample size calculation.

The sample size was based on pharmacokinetic data from a previously published study (28). Twelve subjects in each group (obese and normal weight) allowed the detection of a 19% difference in 12-h drug exposure (area under the concentration-time curve from 0 to 12 h [AUC_0-12_]) and a 16% difference in the maximum concentration of drug in serum (*C*_max_) for the 75-mg dose and a 22% difference in AUC_0-12_ and 24% in *C*_max_ for the 150-mg dose. This assumed a 2-sided significance level of 5% with 90% power. For the comparison of an increase in AUC_0-12_ and *C*_max_ due to the increased dose, differences of 14% for AUC_0-12_ and *C*_max_ could be detected with 80% power, assuming between-subject variability of 20% and a moderate correlation of 0.6 between 2 measurements taken in the same subject.

### Study subjects.

Twelve healthy male and female obese subjects and 12 healthy nonobese subjects who met all of the study inclusion and none of the exclusion criteria were enrolled and hospitalized in the pharmacokinetics unit at the Hospital for Tropical Diseases, Faculty of Tropical Medicine, Mahidol University, Bangkok, Thailand. The study took place from July to October 2010. They were informed about the study purpose, procedures, and risks of participating. All participants provided signed informed consent before screening and enrollment. The study protocol was approved by the Faculty of Tropical Medicine Ethics Committee (FTM-EC) and the Oxford Tropical Research Ethics Committee (OXTREC).

### Inclusion criteria.

The inclusion criteria were healthy males or females, 18 to 60 years of age, who were nonalcoholic (defined as no alcohol consumption within 6 months of screening). The BMIs were ≥30 kg/m^2^ and 18 to 24.9 kg/m^2^ for inclusion in the obese and nonobese groups, respectively. Only obese subjects who did not report major fluctuations in weight in the last 6 months were included. Female subjects with childbearing potential who had not been surgically sterilized were required to use effective methods of contraception during the study period and until 4 weeks after the last dose of the study drug.

### Exclusion criteria.

The exclusion criteria were hypersensitivity to oseltamivir; clinical illness; personal or family history of cardiac disease; an abnormal serum transaminase enzyme level (≥1.5 times the upper limit of normal); estimated creatinine clearance of <70 ml/min by the Cockcroft-Gault equation; HIV-1; hepatitis B surface antigen or hepatitis C antibody positive; positive urine pregnancy test or lactation; abnormal electrocardiogram, especially with a corrected QT interval (QTc) longer than 450 ms using Bazett's formula; other concomitant medication; administration of influenza vaccine or any other antiviral influenza medications within 14 days prior to enrollment; or inability to comply with the protocol procedures.

### Drug analysis.

Oseltamivir and oseltamivir carboxylate were quantified in venous plasma by liquid chromatography (LC) coupled with tandem mass spectrometry (MS-MS) detection as described previously (29). Three replicates of quality control samples at three different concentrations were analyzed within each batch for performance monitoring. Total accuracies were 2.76%, 2.85%, and 2.52% at 3, 20, and 150 ng/ml, respectively, for oseltamivir and 2.42%, 2.50%, and 1.96% at 30 ng/ml, 400 ng/ml, and 4,000 ng/ml, respectively, for oseltamivir carboxylate. The assay used 50 μl of plasma with stable-isotope-labeled internal standards. The lower limits of quantification (LLOQ) were set to 1.0 ng/ml and 10 ng/ml for oseltamivir and oseltamivir carboxylate, respectively.

### Pharmacokinetic analysis.

Individual concentration-time data were evaluated using a noncompartmental approach in WinNonlin version 5.3 (Pharsight Corporation, CA). Concentration data below the LLOQ before the first observed nonzero concentration were replaced with zero. Other LLOQ concentrations were omitted. Total exposure up to the last measured concentration (AUC_0-LAST_) was calculated using the linear trapezoidal method for ascending concentrations and the logarithmic trapezoidal method for descending concentrations. Drug exposure was extrapolated from the last observed concentration (*C*_LAST_) to infinity by *C*_LAST_/λ_*Z*_ (where λ_*Z*_ is the apparent terminal elimination rate constant) for each individual subject to compute the total drug exposure (AUC_0-∞_). The terminal elimination half-life (*t*_1/2_) was estimated by log linear regression of the observed concentrations in the terminal elimination phase. *C*_max_ and time to maximum concentration (*T*_max_) were taken directly from the observed data. The apparent volume of distribution (*V_Z_*/*F*) and oral clearance (CL/*F*) were computed individually according to standard procedures in the software. Complete *in vivo* conversion of oseltamivir into oseltamivir carboxylate was assumed, and the administered dose of oseltamivir carboxylate was calculated using the relative difference in molecular weight.

Individual pharmacokinetic parameter estimates were compared between standard-dose (75-mg) and high-dose (150-mg) administration using the Wilcoxon matched-pairs signed rank test in STATA v.11.1 (StataCorp, TX). Pharmacokinetic parameter estimates were compared between obese and nonobese subjects using the nonparametric Mann-Whitney test in STATA. Individual (standard dose/high dose) ratios and mean (obese/nonobese) ratios of parameter estimates were calculated and summarized to illustrate trends and the direction of significant differences.

## RESULTS

### Study subjects.

Fifteen obese and 16 nonobese subjects were screened. Twelve obese and 12 nonobese subjects who met the study criteria were enrolled. All completed both study regimens. A summary of the baseline characteristics is shown in Table 1. There were 2 men and 10 women in the obese group and 4 men and 8 women in the control group. The median BMIs (range) were 33.8 (30.8 to 43.2) and 22.2 (18.8 to 24.2) for obese and nonobese subjects, respectively. The median creatinine clearance and plasma glucose levels were significantly higher (*P* < 0.05) in the obese group than in the nonobese group. All other demographic factors were similar between the two groups.

**TABLE 1:** Baseline subject characteristics

Characteristic	Value^*a*^		*P* value^*b*^
	Obese (*n* = 12)	Nonobese (*n* = 12)	
Male	16.7 (2/12)	33.3 (4/12)	0.640
Age (yr)	37.0 (27.0–46.0)	31.5 (22.0–45.0)	0.173
Body wt (kg)	87.3 (69.6–120.5)	59.1 (51.0–71.7)	**<0.001**
BMI (kg/m^2^)	33.8 (30.8–43.2)	22.2 (18.8–24.2)	**<0.001**
Serum creatinine (mg/dl)	0.8 (0.7–1.1)	0.7 (0.6–1.2)	0.328
Creatinine clearance (ml/min)^*c*^	125.0 (80.6–178.6)	100.0 (76.8–122.8)	**0.013**
Blood glucose (mg/dl)	96.5 (85.0–115.0)	88.5 (83.0–95.0)	**0.005**
Total cholesterol (mg/dl)	200.0 (153.0–250.0)	185.0 (146.0–227.0)	0.470
Triglycerides (mg/dl)	107.5 (65.0–220.0)	86.0(53.0–127.0)	0.105
Albumin (mg/dl)	4.2 (3.6–4.3)	4.3 (3.8–4.7)	0.089

a Values are reported as median (range) except for gender, which is reported as percentage (ratio).

b *P* values were calculated using Fisher's exact test. Boldface indicates statistical significance, *P* < 0.05.

c Creatinine clearance was determined by the Cockcroft-Gault equation.

### Safety results.

One obese subject experienced moderate nausea and vomiting, probably related to the study medication, around 2 h after receiving 75 mg and at 5 h after the 150-mg regimen. One obese volunteer developed mild nausea 20 min after the 75-mg dose. One nonobese volunteer experienced moderate nausea and vomiting 2 h after the 75-mg dose. There were no clinically relevant changes in laboratory parameters or electrocardiograms (from baseline) in the obese or nonobese group.

### Pharmacokinetic results.

**FIG 1:**
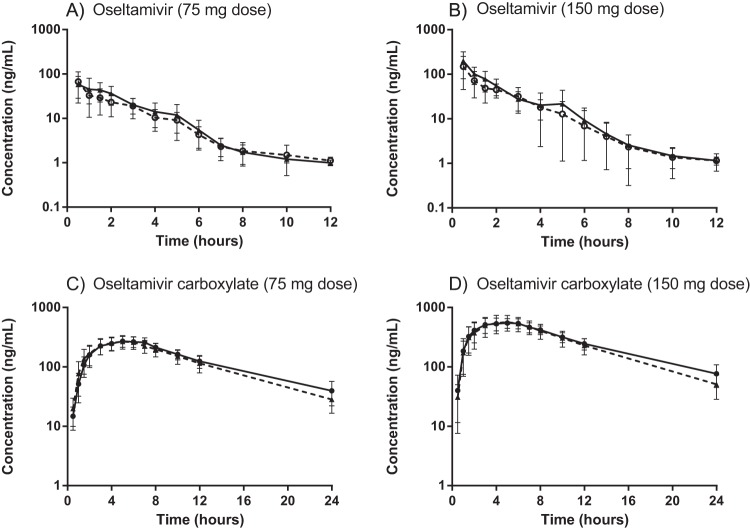
Mean (±standard deviation [SD]) concentration-time profiles of oseltamivir (A and B) and oseltamivir carboxylate (C and D) stratified by dose regimen (75-mg [A and C] or 150-mg [B and D] oseltamivir dose) in nonobese (solid lines) and obese (dashed lines) healthy Thai subjects.

The pharmacokinetic properties of oseltamivir and its active metabolite, oseltamivir carboxylate, were successfully characterized in all subjects at both doses (Table 2 and Fig. 1).

**TABLE 2:** Oseltamivir and oseltamivir carboxylate pharmacokinetic parameters stratified by dose regimen in nonobese and obese healthy Thai volunteers

Parameter^*a*^	75-mg dose			150-mg dose		
	Value [median (range)]		*P* value^*b*^	Value [median (range)]		*P* value^*b*^
	Nonobese subjects (*n* = 12)	Obese subjects (*n* = 12)		Nonobese subjects (*n* = 12)	Obese subjects (*n* = 12)	
Oseltamivir						
*C*_max_ (ng/ml)	74.4 (36.9–150)	78.9 (22.3–148)	0.8625	192 (59.0–394)	153 (46.4–292)	0.204
*T*_max_ (h)	0.500 (0.500–1.50)	0.500 (0.500–3.00)	0.5485	0.500 (0.500–5.00)	0.500 (0.500–5.00)	0.7144
CL/*F* (liters/h)	466 (370–669)	583 (422–922)	**0.0496**	476 (349–869)	631 (359–1,270)	**0.0209**
*V*/*F* (liters)	1,060 (822–2,440)	1,160 (842–3,230)	0.2482	1,110 (767–2,090)	1,190 (722–2,460)	0.5254
*t*_1/2_ (h)	1.41 (1.18–2.88)	1.44 (1.09–2.96)	0.5637	1.55 (1.36–2.52)	1.35 (1.04–1.88)	**0.0243**
AUC_0-LAST_ (h · ng/ml)	159 (111–200)	126 (80.0–176)	**0.0496**	313 (170–425)	237 (116–417)	**0.0209**
AUC_0-∞_ (h · ng/ml)	161 (112–203)	129 (81.3–178)	**0.0496**	315 (172–429)	239 (118–418)	**0.0209**
AUC_0-∞_/dose [(h · ng/ml)/mg]	2.15 (1.49–2.70)	1.72 (1.08–2.37)	**0.0496**	2.10 (1.15–2.86)	1.59 (0.786–2.79)	**0.0209**
Oseltamivir carboxylate						
*C*_max_ (ng/ml)	291 (183–379)	267 (184–402)	0.4703	550 (390–769)	615 (375–888)	0.954
*T*_max_ (h)	6.00 (3.00–7.00)	5.00 (3.00–6.00)	0.13	5.50 (3.00–6.00)	5.00 (3.00–6.00)	0.5903
CL/*F* (liters/h)	19.6 (14.0–25.7)	21.9 (12.8–30.6)	0.204	19.0 (14.6–25.3)	21.4 (12.9–38.3)	0.1489
*V*/*F* (liters)	175 (132–290)	179 (120–278)	0.954	179 (123–276)	155 (110–276)	0.3556
*T*_1/2_ (h)	6.17 (4.26–10.3)	5.99 (4.28–7.10)	0.2727	6.30 (4.72–9.68)	5.21 (4.28–7.42)	**0.0209**
AUC_0-LAST_ (h · ng/ml)	3,020 (2,240–3,870)	2,920 (2,100–4,800)	0.2253	6,310 (4,610–7,820)	6,040 (3,400–9,740)	0.3556
AUC_0-∞_ (h · ng/ml)	3,500 (2,650–4,860)	3,120 (2,230–5,330)	0.204	7,190 (5,380–9,320)	6,360 (3,550–10,600)	0.1489
AUC_0-∞_/dose [(h · ng/ml)/mg]	46.7 (35.3–64.8)	41.6 (29.7–71.1)	0.204	48.0 (35.9–62.1)	42.4 (23.7–70.6)	0.1489

a *C*_max_, maximum observed plasma concentration after oral administration; *T*_max_, observed time to reach *C*_max_; CL, elimination clearance; *V*, apparent volume of distribution; *t_1/2_*, terminal elimination half-life; AUC_0-LAST_, observed area under the plasma concentration-time curve from zero to last observed concentration; AUC_0-∞_, predicted area under the plasma concentration time curve after the last dose from zero to infinity; *F*, oral bioavailability.

b *P* values were calculated using the Mann-Whitney U test. Boldface indicates statistical significance, *P* < 0.05.

Double peak concentrations of oseltamivir were observed in 7 nonobese subjects. Four subjects displayed double peaks at both standard and high dosages, while one and two subjects displayed a double peak after only the standard or only the high dose, respectively. Double peaks of oseltamivir were also observed in 7 obese subjects. Three subjects displayed double peaks at both standard and high dosages, and three and one subject displayed a double peak after only the standard or only the high dose, respectively. Double peaks might be explained by enterohepatic recirculation (30).

### Pharmacokinetics in obese and nonobese subjects.

Elimination clearance of oseltamivir was significantly higher in obese subjects than in nonobese subjects, which resulted in significantly lower (*P* < 0.05) total oseltamivir exposure after both the 75-mg dose and the 150-mg dose (Table 2 and Fig. 1). The median decreases in exposure in obese subjects were 17.9% for the 75-mg dose and 21.8% for the 150-mg dose compared to nonobese subjects. This also resulted in a significantly shorter terminal elimination half-life after the higher, 150-mg dose (*P* = 0.024) but did not reach statistical significance in the lower, 75-mg dose group (*P* = 0.56). Obesity did not have a significant (*P* > 0.05) effect on any other oseltamivir pharmacokinetic parameters.

The same trend of a lower dose-normalized total exposure of oseltamivir carboxylate was observed in obese subjects compared to nonobese subjects (Table 2 and Fig. 1). However, this small difference did not reach statistical significance (*P* = 0.20 after the 75-mg dose and *P* = 0.15 after the 150-mg dose). No pharmacokinetic differences (*P* < 0.05) were observed for oseltamivir carboxylate between obese and nonobese subjects except for a significantly shorter terminal elimination half-life in obese subjects after the higher, 150-mg dose (*P* = 0.021).

### Pharmacokinetics after low (75-mg) and high (150-mg) doses.

**FIG 2:**
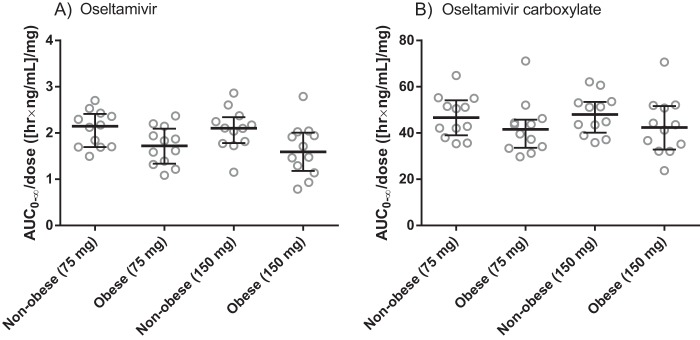
Total dose-normalized exposure of oseltamivir (A) and oseltamivir carboxylate (B) stratified by dose regimen in nonobese and obese healthy Thai subjects. The long horizontal bars indicate median values, and the error bars indicate the interquartile range.

Higher doses of oseltamivir (150 mg) resulted in an expected increase in the maximum concentration and total exposure of both oseltamivir and oseltamivir carboxylate (Table 3 and Fig. 2). Dose-normalized total drug exposures were not significantly different between high and low doses in any group, which confirms dose-linear pharmacokinetics in the studied dose range.

**TABLE 3:** Dose linearity of oseltamivir and oseltamivir carboxylate pharmacokinetics in nonobese and obese healthy Thai volunteers

Parameter	Individual ratio (150-mg dose/75-mg dose)			
	Nonobese subjects (*n* = 12)		Obese subjects (*n* = 12)	
	Median (range)	*P* value^*a*^	Median (range)	*P* value^*a*^
Oseltamivir				
*C*_max_ (ng/ml)	2.41 (1.36–5.92)	**0.0022**	2.06 (0.743–6.27)	**0.0121**
*T*_max_ (h)	1.00 (0.333–10.0)	0.5816	1.00 (0.167–5.00)	0.6816
CL/*F* (liters/h)	0.982 (0.904–1.30)	0.8139	1.13 (0.789–2.02)	0.2393
*V*/*F* (liters)	1.09 (0.483–1.77)	0.6379	0.999 (0.407–2.20)	0.7537
*t*_1/2_ (h)	1.02 (0.490–1.85)	0.5829	0.917 (0.451–1.16)	**0.0499**
AUC_0-LAST_ (h · ng/ml)	2.06 (1.54–2.23)	**0.0022**	1.80 (0.991–2.55)	**0.0029**
AUC_0-∞_ (h · ng/ml)	2.04 (1.54–2.21)	**0.0022**	1.78 (0.991–2.54)	**0.0029**
AUC_0-∞_/dose [(h · ng/ml)/mg]	1.02 (0.770–1.11)	0.9375	0.888 (0.496–1.27)	0.3078
Oseltamivir carboxylate				
*C*_max_ (ng/ml)	2.09 (1.59–2.44)	**0.0022**	2.15 (1.54–2.59)	**0.0022**
*T*_max_ (h)	0.929 (0.600–1.33)	0.0519	1.00 (0.800–1.29)	0.6219
CL/*F* (liters/h)	0.978 (0.885–1.07)	0.0597	0.986 (0.855–1.44)	0.3465
*V*/*F* (liters)	0.958 (0.844–1.09)	0.3465	0.906 (0.776–1.32)	**0.0342**
*t*_1/2_ (h)	0.995 (0.876–1.11)	0.9375	0.913 (0.818–1.04)	**0.0096**
AUC_0-LAST_ (h · ng/ml)	2.06 (1.92–2.22)	**0.0022**	2.05 (1.40–2.40)	**0.0022**
AUC_0-∞_ (h · ng/ml)	2.04 (1.87–2.26)	**0.0022**	2.03 (1.39–2.34)	**0.0022**
AUC_0-∞_/dose [(h · ng/ml)/mg]	1.02 (0.935–1.13)	0.1167	1.01 (0.695–1.17)	0.4328

a *P* values were calculated using the Wilcoxon signed rank test. Boldface indicates statistical significance, *P* < 0.05.

## DISCUSSION

The subjects of this study were predominantly female, corresponding to the geographic distribution of the obese population in the study area (21, 24–26, 31, 32). The study showed that obesity had an impact in reducing the exposure of oseltamivir but did not affect the pharmacokinetic characteristics of oseltamivir carboxylate, the main active metabolite that inhibits influenza virus replication. The significantly lower exposure of oseltamivir in obese than in nonobese subjects is likely to be of no clinical relevance, since oseltamivir is a prodrug and not therapeutically active. The pharmacokinetic properties of oseltamivir and oseltamivir carboxylate in both obese and nonobese subjects in this study were dose linear and proportionate for 75-mg and 150-mg regimens, which is consistent with earlier studies (33–38). This supports current dose recommendations.

We assumed no ethnopharmacological differences among Asian and Caucasian patients when comparing the results in this study to the available literature (19, 20). This is supported further by a study that showed similar pharmacokinetics of oseltamivir in healthy Japanese and Caucasian volunteers (39). Two recent publications explored the pharmacokinetic properties of oral oseltamivir in healthy obese subjects. Pai and Lodise reported a study in 21 healthy morbidly obese subjects with a very high mean BMI of 46 kg/m^2^ (19). The pharmacokinetic estimates after the first dose of 75 mg were in agreement with our data. The investigators also concluded that the data on morbidly obese subjects were consistent with findings in nonobese subjects based on comparisons with historical data and proposed that dose adjustment in the morbidly obese was not necessary. Thorne-Humphrey and colleagues conducted a pharmacokinetic study in which 10 healthy morbidly obese Caucasian subjects with a median BMI of 46 kg/m^2^ were compared with 10 healthy nonobese subjects (BMI < 30 kg/m^2^). The pharmacokinetic results in this study were comparable to data presented in our study (20).

Recently, Ariano and colleagues reported similar findings in a clinical pharmacokinetic study in which critically ill patients across a weight range of 50 to 200 kg with a high mean BMI of 36 kg/m^2^ who had suspected or confirmed influenza received either 75 mg or 150 mg of oseltamivir twice daily. The pharmacokinetic parameters of oseltamivir carboxylate in patients with normal renal function were close to the data for the healthy obese subjects in our study (10). The study also found no correlation between body weight and oseltamivir carboxylate exposure or volume of distribution.

A number of studies have shown that the trough levels of oseltamivir carboxylate exceeded the range of drug concentrations required to inhibit 50% of the neuraminidase activities (IC_50_ range) of various types of influenza virus, including pandemic H1N1 2009 (IC_50_ range, 0.01 to 69.2 nM) in both obese volunteers and obese patients receiving 75 mg or 150 mg twice daily for 5 days (5, 6, 10, 19, 20, 40–42). Ariano and colleagues also showed that the average plasma oseltamivir carboxylate concentration was much higher than the IC_50_ of the 2009 H1N1 influenza virus strain in patients with natural influenza virus infections (10). Obviously, we cannot predict the accumulation of drug over time from a single dose study analyzed with a model-independent analysis. However, the median (range) observed oseltamivir carboxylate concentrations were 397 (255 to 731) nM and 422 (306 to 668) nM at 12 h after a single 75-mg dose in obese and nonobese subjects, respectively, suggesting this dose is more than adequate to reach the IC_50_s reported in previously published studies.

The rapid increase in obesity has a significant public health impact and is likely to be a burden on health services in the near future. The unusual clinical manifestations and higher morbidity and mortality among the obese during the 2009 H1N1 pandemic needs further investigation and cannot be explained by a difference in the pharmacokinetics of the antiviral medication. Further research into host defense mechanisms and organ functional alterations, including the pharmacodynamics of oseltamivir in obese natural influenza patients, is warranted.

Standard, unadjusted doses of oseltamivir provide similar exposures to oseltamivir carboxylate, the active metabolite of oseltamivir, in obese and nonobese subjects. Further studies are needed to evaluate why obese patients with influenza are at higher risk of severe disease than nonobese patients.
